# Cold waves and fine particulate matter in high-altitude Chinese cities: assessing their interactive impact on outpatient visits for respiratory disease

**DOI:** 10.1186/s12889-024-18896-x

**Published:** 2024-05-22

**Authors:** Zhenxu Ning, Shuzhen He, Xinghao Liao, Chunguang Ma, Jing Wu

**Affiliations:** 1 Department of Public Health, Faculty of Medicine, Qinghai University, Xining, China; 2 Xining Centre for Disease Control and Prevention, Xining, China

**Keywords:** Cold wave, Fine particulate matter, Respiratory disease, Air pollution, Climate change

## Abstract

**Background:**

Extreme weather events like heatwaves and fine particulate matter (PM_2.5_) have a synergistic effect on mortality, but research on the synergistic effect of cold waves and PM_2.5_ on outpatient visits for respiratory disease, especially at high altitudes in climate change-sensitive areas, is lacking.

**Methods:**

we collected time-series data on meteorological, air pollution, and outpatient visits for respiratory disease in Xining. We examined the associations between cold waves, PM_2.5_, and outpatient visits for respiratory disease using a time-stratified case-crossover approach and distributional lag nonlinear modeling. Our analysis also calculated the relative excess odds due to interaction (REOI), proportion attributable to interaction (AP), and synergy index (S). We additionally analyzed cold waves over time to verify climate change.

**Results:**

Under different definitions of cold waves, the odds ratio for the correlation between cold waves and outpatient visits for respiratory disease ranged from 0.95 (95% CI: 0.86, 1.05) to 1.58 (1.47, 1.70). Exposure to PM_2.5_ was significantly associated with an increase in outpatient visits for respiratory disease. We found that cold waves can synergize with PM_2.5_ to increase outpatient visits for respiratory disease (REOI > 0, AP > 0, S > 1), decreasing with stricter definitions of cold waves and longer durations. Cold waves’ independent effect decreased over time, but their interaction effect persisted. From 8.1 to 21.8% of outpatient visits were due to cold waves and high-level PM_2.5_. People aged 0–14 and ≥ 65 were more susceptible to cold waves and PM_2.5_, with a significant interaction for those aged 15–64 and ≥ 65.

**Conclusion:**

Our study fills the gap on how extreme weather and PM_2.5_ synergistically affect respiratory disease outpatient visits in high-altitude regions. The synergy of cold waves and PM2.5 increases outpatient visits for respiratory disease, especially in the elderly. Cold wave warnings and PM_2.5_ reduction have major public health benefits.

**Supplementary Information:**

The online version contains supplementary material available at 10.1186/s12889-024-18896-x.

## Introduction

During the past few years, the global climate change process has intensified, with frequent occurrences of extreme weather events such as heatwaves and cold waves imposing a heavy burden on global public health and socio-economics [1–4]. Meanwhile, the issue of air pollution persists, and the combined impact of climate change and air pollution exacerbates the health burden on the people [5, 6]. Cold waves, not only increase the body’s stress response and affect physical and cognitive functions but can also induce changes in airway function and bronchoconstriction [7–9], thereby exacerbating respiratory disease, such as asthma and chronic obstructive pulmonary disease [10, 11]. Additionally, the hypoxic and low-pressure environment in high-altitude areas can trigger various diseases by stimulating hypoxia-inducible factors, enhancing inflammatory responses, and damaging mitochondrial functions, with the impact on health possibly becoming more pronounced during cold wave periods [12–14]. Fine particulate matter (PM_2.5_), as a major component of air pollution, is widely recognised for its aggravating effects on respiratory disease [15]. Although previous studies have focused on the individual effects of cold waves and high PM_2.5_ concentrations, research on their synergistic effects, especially in winter when both are commonly present, remains relatively scarce. While current research has shown synergistic effects between extreme temperatures and PM_2.5_, most of these studies have concentrated on the impacts of extreme heat [16–18]. Research on the synergistic effects of cold waves and PM_2.5_, particularly their impact on outpatient visits for respiratory disease, is almost nonexistent. As the capital city of a high-altitude province in China, Xining’s long heating period in winter, which lasts for half a year, and the prolonged cold period may exacerbate air pollution and health problems [19, 20], especially the negative impact on respiratory health.

To fill this gap, this study explored the relationship between exposure to cold waves and PM_2.5_ and outpatient visits for respiratory disease, quantitatively assessed their interactive effects on respiratory disease visits, and estimated the corresponding excess visit rates and numbers of visitors. We also conducted stratified analyses to explore potential vulnerable groups. In addition, we considered the exposure to cold waves and PM_2.5_ in different time periods to assess their specific impacts in the context of climate change in plateau areas.

## Materials and methods

### Study sites

**Fig. 1:**
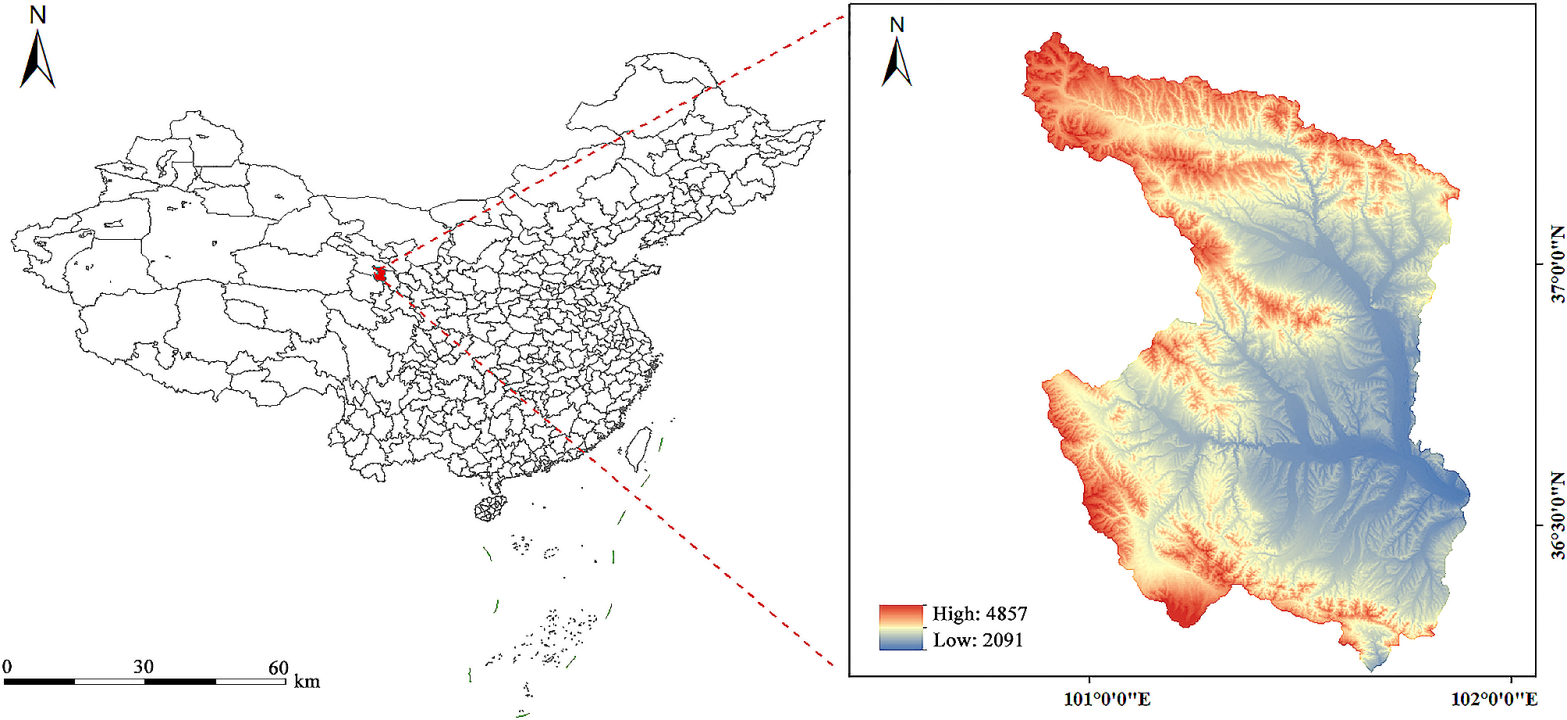
Location and altitude range of Xining in China

Xining, situated in the Qinghai-Tibet Plateau’s northeastern sector, spans altitudes from 2091 to 4857 m (Fig. 1). The terrain is higher in the southwest and lower in the northeast. This study covers five districts of Xining City, including Chengdong, Chengzhong, Chengxi, Chengbei, and Huangzhong, as well as two counties, Huangyuan and Datong Hui and Tu Autonomous County. The total population of Xining is approximately 2.468 million, accounting for about 42% of Qinghai Province’s population. The climate belongs to the cold temperate category of high mountains and plateaus. The winter is cold and prolonged, with an average annual temperature of about 6℃, and the lowest temperature can reach − 18.9℃.

### Data collection and cold wave definitions

We obtained outpatient visits data for respiratory disease (ICD-10: J00-J99) from the majority of hospitals in Xining city, covering the period from January 1, 2016, to December 31, 2021. The data included information on age, gender, disease diagnosis, and codes based on the International Classification of Disorders, Tenth Revision (ICD-10). The data were classified according to sex (female and male) and age categories (0–14 years, 15–64 years, and ≥ 65 years). The Qinghai Provincial Meteorological Bureau produced meteorological data, encompassing average daily temperatures and humidity. Five national control monitoring stations in the Xining city urban area provided average daily concentrations of air pollutants PM_2.5_, SO_2_, CO, O_3_, NO_2_, and PM_10_. In addition, data from the China Air Quality Online Monitoring and Analysis Platform were used to supplement the missing pollutant data (https://www.aqistudy.cn/). To address air monitoring data with a missing rate of less than 5%, we employed multiple imputation techniques to complete the data [21]. Subsequently, we utilised the daily average values from several monitoring stations as the data for atmospheric pollutants. There was a complete absence of any missing meteorological data, and the values exhibited logical consistency.

Within this study, cold waves were characterised as daily average temperatures falling below specific percentiles (2.5th, 5th, 7.5th, or 10th) and persisting for a minimum of 2 to 4 consecutive days, based on previous studies [22, 23]. For instance, 7.5th -2D denotes a cold wave defined as at least 2 consecutive days with daily apparent temperature at or below the 7.5th percentile. Furthermore, based on the equal time span and variation in cold wave frequency, we considered cold wave and PM_2.5_ exposure at different time periods, aiming to gain insight into the impact of climate change on the plateau region.

### Statistical analysis

We utilised a time-stratified case-crossover approach and employed conditional logistic regression to quantitatively examine the correlation between cold waves, PM_2.5_, and outpatient visits for respiratory disease [17, 18]. In the case-crossover design, each study subject acts as their own control. The visit date was designated as the case day, while other dates in the same year, month, and day of the week as the case day were designated as control days. Each case period was matched with three or four control periods before or after the case period to control for long-term trends, seasonal trends, and day-of-week effects. For instance, if a participant visited on a Tuesday in August 2021, that specific day would be considered the case day, while the remaining Tuesdays in that month would serve as control days.

On the basis of the above, we used a distributed lag non-linear model (DLNM) to calculate the exposure-response and lag-response correlation between cold waves, PM_2.5_, and outpatient visits for respiratory disease [24]. The linear function was employed to represent the exposure-response relationship [25], while the natural spline (ns) with 3 degrees of freedom (df) was utilised to suit the lag-response correlation. According to previous studies, the lag of cold wave was typically 21 days [26] and the lag of PM_2.5_ was typically 7 days [27]. Based on previous research, the natural spline (ns) with 3 df was used to control for the confounding effects of relative humidity [28]. The expression is as follows:

$$\begin{aligned}\varvec{l}\varvec{o}\varvec{g}\left(\varvec{E}\right(\varvec{Y}\left)\right)&=\varvec{\alpha }+\varvec{c}\varvec{b}({\varvec{C}\varvec{S}}_{\varvec{i}}/{\varvec{P}\varvec{M}}_{\varvec{i}},\varvec{l}\varvec{a}\varvec{g})\\&+\varvec{n}\varvec{s}(\varvec{r}\varvec{h},3)+\varvec{s}\varvec{t}\varvec{r}\varvec{a}\varvec{t}\varvec{u}\varvec{m}+\varvec{v}\varvec{a}\varvec{c}\varvec{a}\varvec{t}\varvec{i}\varvec{o}\varvec{n}\end{aligned}$$

Where E(Y) is the expected number of daily outpatient visits; α is the intercept; cb(CS) and cb(PM) are cross-basis functions for cold waves and PM_2.5_, used to examine lag effects; stratum is a time-stratification variable, used to control for long-term trends, seasonal changes, and other time-related factors; ns (rh, 3) is a natural cubic spline of relative humidity with 3 df; vacation is a binary variable used to control for Chinese holidays.

In order to further examine the interactive effects of exposure to cold waves and PM_2.5_ on outpatient visits for respiratory disease, we categorised PM_2.5_ exposure into two categories (low concentration: ≤37.5 µg/m^3^, high concentration: >37.5 µg/m^3^) based on the World Health Organisation’s 2021 Air Quality Guidelines Interim Target 3 for PM_2.5_ [29, 30]. We then created a new variable with 4 levels to represent different combinations of exposure to cold waves and PM_2.5_. These levels include: (1) no-cold wave and low-level PM_2.5_ (level 1), (2) cold wave and low-level PM_2.5_ (level 2), (3) no-cold wave and high-level PM_2.5_ (level 3), and (4) cold wave and high-level PM_2.5_ (level 4), with level 1 serving as the reference group [17]. The assessment of this effect involved the inclusion of this variable in a conditional logistic regression model, utilising three measures: the relative excess odds ratio due to interaction (REOI), the proportion attributable to interaction (AP), and the synergy index (S), which quantified the proportion of the effect that can be attributed to interaction. The formulas below were used to calculate the proportions of joint effects resulting from interactions and the proportions of joint effects compared to individual effects for these 3 indicators [31–33]:

$$\begin{aligned}\varvec{R}\varvec{E}\varvec{O}\varvec{I}&=({\varvec{O}\varvec{R}}_{11}-1)-({\varvec{O}\varvec{R}}_{10}-1)-({\varvec{O}\varvec{R}}_{01}-1)\\&={\varvec{O}\varvec{R}}_{11}-{\varvec{O}\varvec{R}}_{10}-{\varvec{O}\varvec{R}}_{01}+1\end{aligned}$$

$$\varvec{A}\varvec{P}=\frac{\varvec{R}\varvec{E}\varvec{O}\varvec{I}}{{\varvec{O}\varvec{R}}_{11}}$$

$$\mathbf{S}=\frac{{\mathbf{O}\mathbf{R}}_{11}-1}{({\mathbf{O}\mathbf{R}}_{10}-1)+({\mathbf{O}\mathbf{R}}_{01}--1)}$$

Where OR_10_, OR_01_, and OR_11_ correspond to levels 2, 3, and 4, respectively, with relation to level 1 (where OR_00_ = 1). When REOI = 0, AP = 0, and S = 1, it means that there is no interaction between the cold wave and PM_2.5_ on respiratory disease visits. On the other hand, when REOI > 0, AP > 0, and S > 1, it indicates that the combined effect of the cold wave and PM_2.5_ on respiratory disease visits is greater than the sum of the effects of individual exposures, which is known as a synergistic effect. Conversely, when REOI < 0, AP < 0, and S < 1, it means that the combined effect is smaller than the sum of the individual effects of the cold wave and PM_2.5_. The delta approach was employed to get the 95% confidence interval (CI) associated with the three indicators [34].

We assessed the independent effects of cold waves and PM_2.5_ on respiratory disease visits and their interactions by disaggregating by sex and age in order to identify potentially vulnerable groups. We utilized two-sample Z-tests to examine whether there were differences in stratum-specific effect estimates for each stratification variable.

$$\varvec{Z}=\frac{{\varvec{\beta }}_{\varvec{m}\varvec{a}\varvec{l}\varvec{e}}-{\varvec{\beta }}_{\varvec{f}\varvec{e}\varvec{m}\varvec{a}\varvec{l}\varvec{e}}}{\sqrt{{\varvec{S}\varvec{E}}_{\varvec{m}\varvec{a}\varvec{l}\varvec{e}}^{2}-{\varvec{S}\varvec{E}}_{\varvec{f}\varvec{e}\varvec{m}\varvec{a}\varvec{l}\varvec{e}}^{2}}}$$

where β represents a particular point estimate in a conditional logistic regression model; SE is the standard error associated with each β.

To verify the reliability of the findings, we modified the degrees of freedom for relative humidity, increasing it from 3 to 6, and changed the linear function (fun=“lin”) of the crossbase of PM_2.5_ to a nonlinear function (fun=“ns”). Furthermore, we incorporated individual air pollutants (NO_2_, CO, SO_2_, and O_3_) as well as combined air pollutants (PM_2.5_ and O_3_) into the model as distinct variables. The sensitivity studies limited the heating period (October 15 to April 15) in Xining City. Additionally, to observe the interference of the COVID-19 pandemic and variations in RR across different time periods, we categorized the study period as follows: 2016–2018, 2016–2019, 2019–2021, and 2020–2021. The percent excess risk was calculated as [(exp[β]-1)]. The statistical analyses in this study were mostly conducted using R software (version 4.3.1).

## Results

During the study period, the average daily temperature and average daily relative humidity in Xining City were 6.4 ± 9.2 (°C) and 71.7 ± 16.2 (%), respectively. Additionally, the average daily concentrations of PM_2.5_, SO_2_, NO_2_, CO, and O_3_ were 40.2 ± 27.9 µg/m^3^, 20.0 ± 13.1 µg/m^3^, 39.3 ± 16.1 µg/m^3^, 1.4 ± 0.8 mg/m^3^, and 93.3 ± 33.4 µg/m^3^. The overall number of outpatient visits for respiratory disease in the population amounted to 393,185 cases from 2016 to 2021 (Table S1). A low to moderate correlation existed between the average daily temperature and other variables (*p* < 0.05) (Figure S2). Among these variables, the correlation between PM_2.5_ and PM_10_ was relatively strong (*p* < 0.05), with a correlation coefficient greater than 0.8. However, the correlation between O_3_ and relative humidity was minimal (*p* > 0.05).

Table 1 shows the number of outpatient visits for respiratory disease in Xining City at different exposure levels from 2016 to 2021. Based on the 7.5th-2D definition (where “7.5th” refers to a daily average temperature below the 7.5th percentile threshold, and “2D” refers to the condition lasting at least two consecutive days), there were a total of 163 days identified with cold waves. Out of these days, 40,238 subjects (10.2%) were observed. The bulk of subjects, 352,947 in total, were observed on days without cold waves. Out of the total number of visits for respiratory disease, 87.3% (35,110) happened during a period of both cold waves and high PM_2.5_ concentrations. Additionally, 11.8% (5,128) of respiratory disease visits occurred during a period of both cold waves and low PM_2.5_ concentrations. Overall, the frequency of respiratory disease visits fell as the temperature thresholds were lowered and the cold wave days lasted longer. Given these preliminary results, we used the definition group of 7.5th-2D to 7.5th-4D for stratified analysis and sensitivity analysis because, in this definition group, the number of cold wave days and the outpatient visits for respiratory disease were relatively concentrated [35, 36].

**Table 1:** Cold wave days with different cold wave definitions and corresponding outpatient visits for respiratory disease in Xining from 2016 to 2021

Definition	Days	Outpatient visits for respiratory disease(%)		
		Overall	With low-level PM_2.5_	With high-level PM_2.5_
10th2D	201	50,511	5,983(11.8)	44,528(88.2)
10th3D	182	45,750	5,607(12.3)	40,143(87.7)
10th4D	164	40,938	4,269(10.4)	36,669(89.6)
7.5th2D	163	40,238	5,128(12.7)	35,110(87.3)
7.5th3D	142	36,532	4,301(11.8)	32,231(88.2)
7.5th4D	119	30,108	3,092(10.3)	27,016(89.7)
5th2D	98	23,981	2,469(10.3)	21,512(89.7)
5th3D	81	18,506	2,358(12.7)	16,148(87.3)
5th4D	63	14,324	1,819(12.7)	12,505(87.3)
2.5th2D	46	10,023	1,713(17.1)	8,310(82.9)
2.5th3D	40	8,838	1,713(19.4)	7,125(80.6)
2.5th4D	31	7,208	1,220(16.9)	5,988(83.1)

**Fig. 2:**
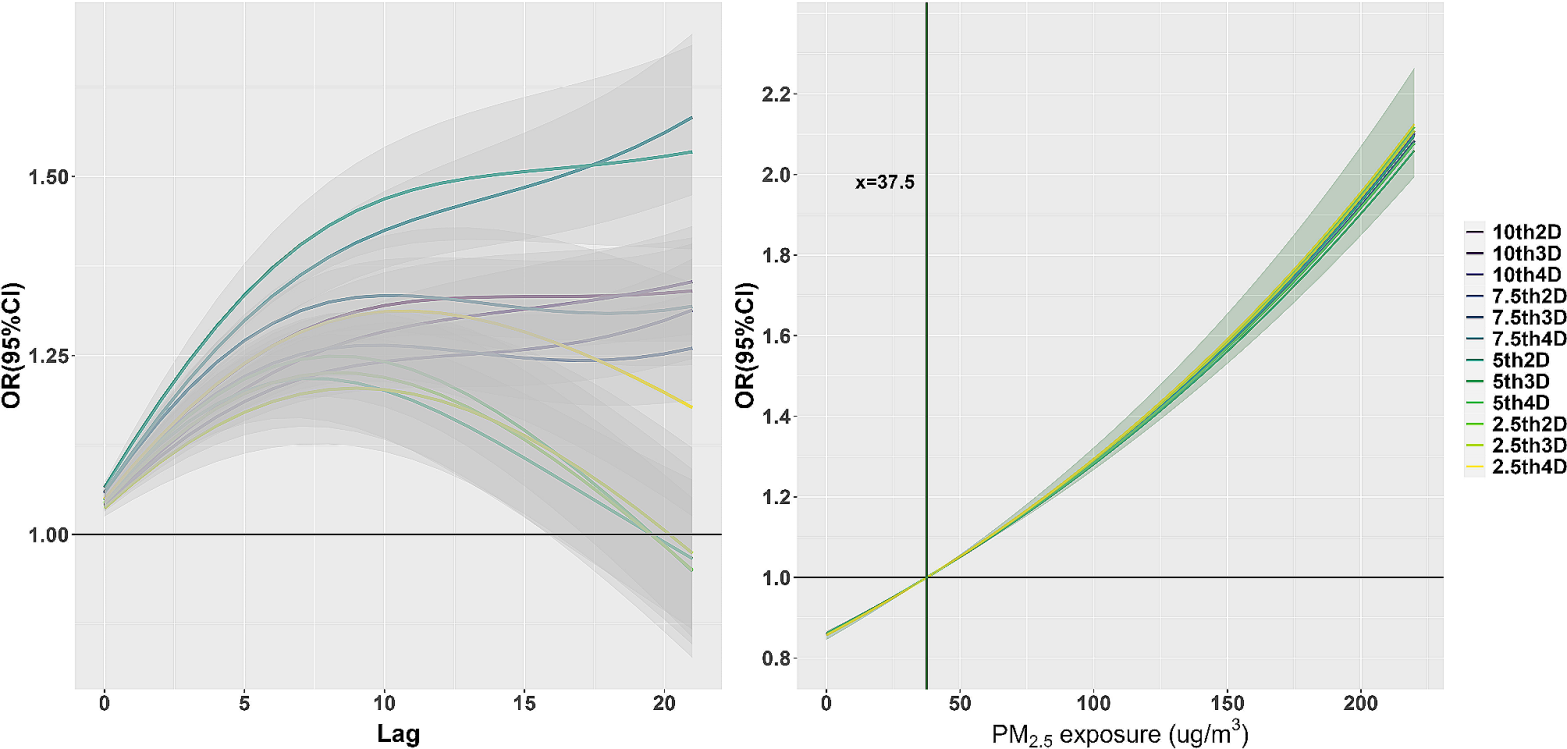
Association of exposure to cold waves and PM_2.5_ with outpatient visits for respiratory disease: **A** OR (95% CI) of outpatient visits for respiratory disease associated with exposure to cold waves with a lag of 0–21 days; **B**: Exposure-response curves of exposure to PM_2.5_ and outpatient visits for respiratory disease with a lag of 0–7 days, adjusted to include different cold wave definitions

Figure 2A depicts the correlation between cold wave exposure and visits to respiratory disease. We observed that exposure to cold waves was significantly associated with increased odds of respiratory disease clinic visits. Using the 10th-2D to define cold waves, the odds ratio (OR) for exposure to cold waves was 1.308 (95%CI:1.258,1.360) (*p* < 0.05), indicating a 30.8% (25.8,36.0) increased risk of clinic visits. The OR decreased gradually as the definition of cold waves became more stringent, possibly due to individuals adopting precautionary measures.

**Table 2:** Additive interaction of exposure to cold waves and PM_2.5_ on outpatient visits for respiratory disease

Definition	Additive interaction		
	REOI(95%CI)	AP(95%CI)	S(95%)
10th2D	0.171(0.135,0.207)	0.140(0.136,0.145)	1.163(1.121,1.205)
10th3D	0.144(0.107,0.182)	0.119(0.115,0.123)	1.135(1.093,1.176)
10th4D	0.152(0.111,0.193)	0.128(0.123,0.133)	1.146(1.097,1.195)
7.5th2D	0.120(0.081,0.159)	0.103(0.099,0.107)	1.115(1.071,1.159)
7.5th3D	0.122(0.078,0.166)	0.102(0.098,0.107)	1.114(1.068,1.160)
7.5th4D	0.135(0.087,0.183)	0.115(0.110,0.120)	1.130(1.077,1.183)
5th2D	0.046(-0.007,0.098)	0.042(0.040,0.044)	1.044(0.991,1.096)
5th3D	0.043(-0.010,0.097)	0.040(0.038,0.042)	1.042(0.987,1.097)
5th4D	0.082(0.021,0.143)	0.074(0.070,0.078)	1.080(1.015,1.145)
2.5th2D	0.079(0.009,0.148)	0.069(0.065,0.073)	1.074(1.009,1.138)
2.5th3D	0.047(-0.025,0.118)	0.042(0.039,0.045)	1.044(0.981,1.107)
2.5th4D	0.071(-0.013,0.154)	0.063(0.059,0.068)	1.067(0.989,1.146)

Figure 2B depicts the correlation between exposure to PM_2.5_ and the frequency of outpatient visits for respiratory disease. When different definitions of “cold wave” were added to the model to make it more accurate, the odds of respiratory disease outpatient visits consistently went up when PM_2.5_ levels were higher.

Figure S2 and Table 2 show the interactive impact of being exposed to cold waves and PM_2.5_ on visits related to respiratory disease. As defined by 7th-D3, the OR_10_, OR_01_, and OR_11_ for respiratory disease visits were 1.044 (95% CI:1.002,1.088), 1.025 (1.013,1.037), and 1.192 (1.159, 1.224); REOI, AP, and S were 0.122 (95% CI:0.078,0.165), 0.102 (0.098,0.106), and 1.114 (1.068, 1.160), suggesting significant synergistic effects of exposure to cold waves and PM_2.5_ on respiratory disease visits. Except for 5th-2D and 5th-3D, similar synergistic effects were found when using alternative definitions of cold waves (REOI > 0, AP > 0, and S > 1; *p* < 0.05). In general, there was a decline observed when implementing more stringent temperature thresholds and extending the duration of cold waves.

**Fig. 3:**
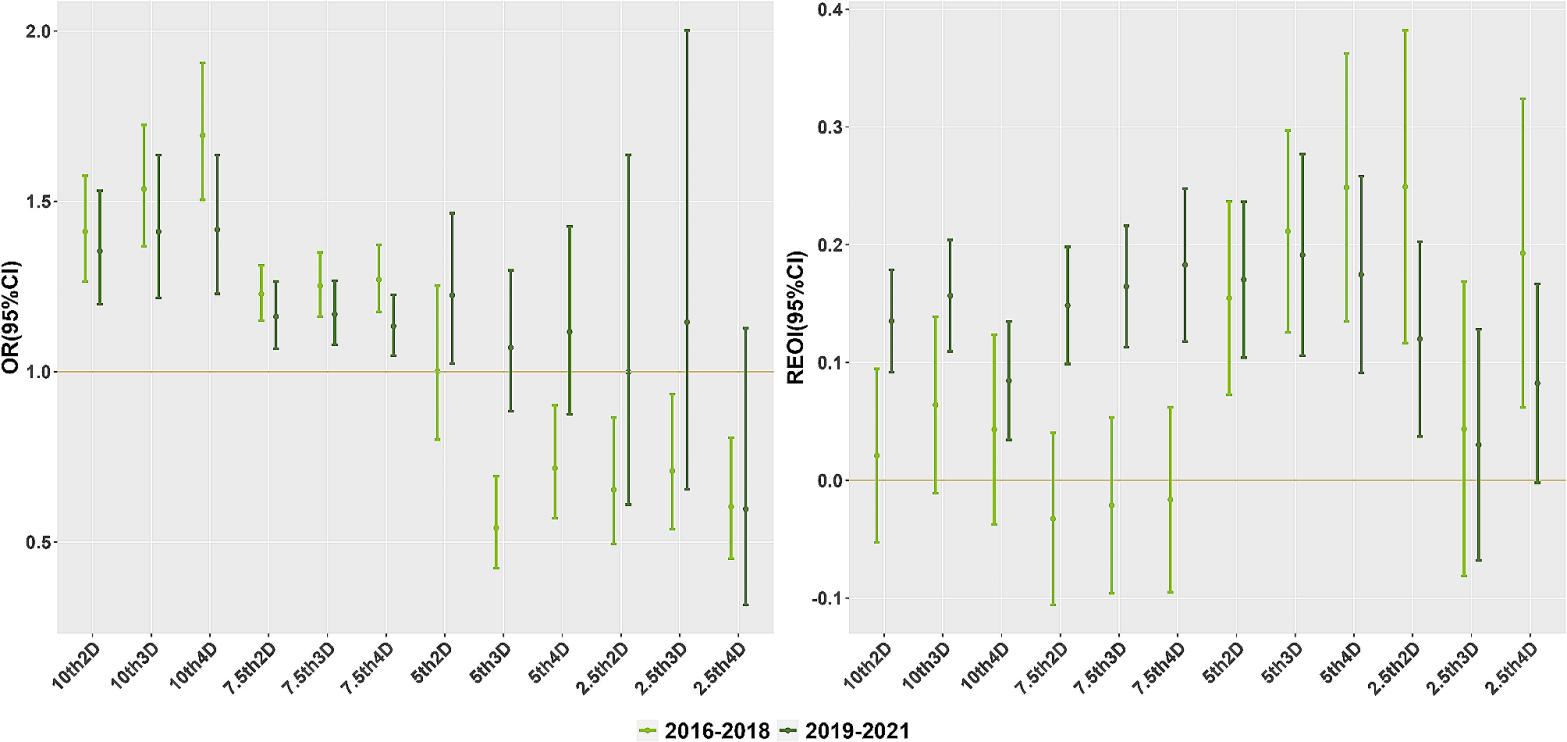
Independent and interactive effects of exposure to cold waves and PM_2.5_ on outpatient visits for respiratory disease at different periods. (Different periods for 2016–2018 and 2019–2021)

Figure 3 illustrate the independent effects and interactions resulting from exposure to cold waves and PM_2.5_ during various time periods on outpatient visits related to respiratory disease. Comparative analysis indicated that the independent impact of cold waves exhibits a decreasing trend. During the period spanning from 2016 to 2018, significant synergistic effects (REOI > 0, AP > 0, and S > 1) of cold waves and PM_2.5_ on respiratory system disease mainly occurred within the defined range of 5th-2D to 2.5th-2D, and no significant synergistic effects were observed in other ranges. Nevertheless, during the period from 2019 to 2021, we observed significant synergistic effects, which may be attributable to the decrease in the independent impact of cold waves.

**Fig. 4:**
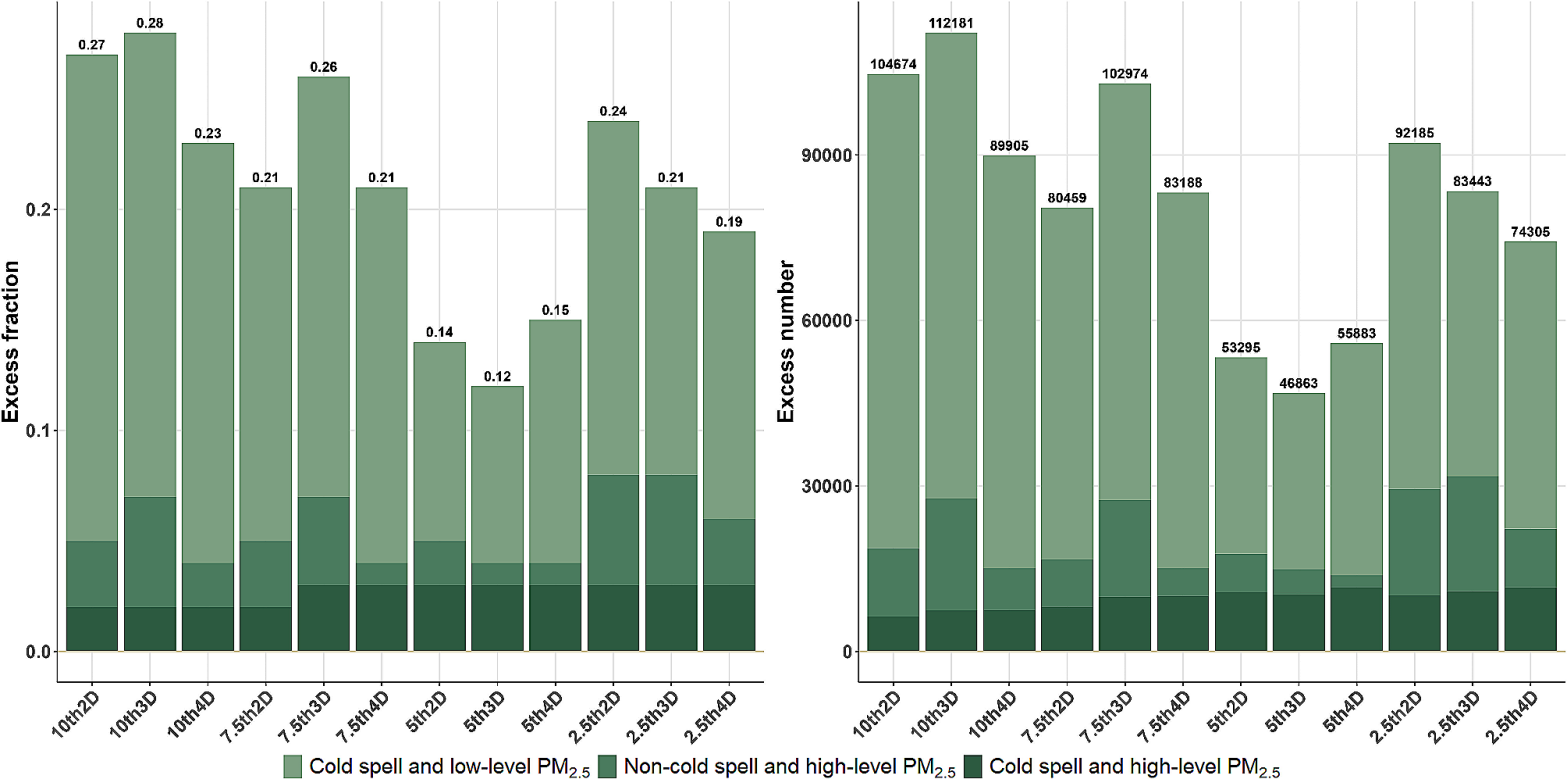
Fraction and number of excess outpatient visits for respiratory disease due to exposure to cold waves and high-level of PM_2.5_

Excess outpatient visit rates and numbers resulting from exposure to cold waves and different concentrations of PM_2.5_ are shown in Fig. 4 and Table S2. The excess rate range under different cold wave definitions spans from 12 to 28%, corresponding to excess outpatient visit numbers ranging from 46,863 to 112,181 cases, respectively. According to the definition of 7.5th-D3, 19.2% of the excess rate was attributed to exposure to cold waves and high concentrations of PM_2.5,_ corresponding to 75,501 cases of outpatient visits for respiratory disease. 2.52% (9,898 cases) was attributed to non-cold wave periods with high levels of PM_2.5_, and 4.47% (17,578 cases) was attributed to cold wave periods with low-level of PM_2.5_. Overall, lower temperature thresholds and longer durations of cold waves were associated with lower excess outpatient visit rates.

The stratified analysis in Table 3 indicates a stronger correlation between cold waves and respiratory disease visit rates in males, children, and the elderly. Meanwhile, the correlation between PM_2.5_ and respiratory disease visit rates varied among age groups (*p* < 0.05). The differences in interaction were statistically significant in the 15–64 age group and those aged ≥ 65 (*p* < 0.05).

**Table 3:** OR and REOI of cold wave and PM_2.5_ exposure on outpatient visits for respiratory disease (stratified by sex, age)

Definition	Sex		Age		
	Male	Female	0–14 years	15–64 years	≥ 65 years
**OR**					
7.5th2D	1.26(1.19,1.34)	1.19(1.12,1.27)	1.26(1.18,1.35)	1.15(1.08,1.23)	1.48(1.30,1.69)^*^
7.5th3D	1.34(1.25,1.42)	1.23(1.15,1.31)	1.37(1.27,1.47)	1.15(1.07,1.23)^*^	1.60(1.39,1.84)^*^
7.5th4D	1.58(1.48,1.70)	1.509(1.40,1.63)	1.70(1.57,1.84)	1.37(1.27,1.48)^*^	1.94(1.66,2.28)^*^
PM_2.5_	1.04(1.03,1.04)	1.04(1.03,1.04)	1.06(1.06,1.07)	1.03(1.02,1.03)^*^	1.05(1.05,1.06)^*^
**REOI**					
7.5th2D	0.139(0.086,0.191)	0.098(0.040,0.156)	0.097(0.036,0.157)	0.024(-0.032,0.081)	0.643(0.518,0.768)^*^
7.5th3D	0.148(0.089,0.207)	0.091(0.026,0.157)	0.078(0.009,0.147)	0.027(-0.036,0.092)	0.675(0.545,0.805)^*^
7.5th4D	0.165(0.101,0.229)	0.099(0.027,0.171)	0.107(0.031,0.182)	0.043(-0.028,0.116)	0.602(0.464,0.740)^*^

^*^p < 0.05,two-sample Z-test estimation

Sensitivity analysis indicates that changes in the relative humidity parameter and cross-base function lead to minor variations in the outcomes (Figure S3-4). This stability was preserved even when accounting for both individual and combined air pollutants. The results concerning the variations in cold waves and PM_2.5_ across different time periods indicate that both factors had already exerted significant impacts prior to the pandemic. However, during the pandemic period, their effects slightly diminished (Figure S5; Table S3). This indicated that COVID-19 and its protective measures may have influenced the health effects of these environmental factors, but this influence was positive. It suggests that when assessing the impact of environmental factors on health, the interference of large-scale public health events needs to be considered. Such outcomes from the sensitivity analysis highlighted the reliability of our research findings.

## Discussion

We examined the correlation between cold waves and PM_2.5_ levels in the high-altitude city of Xining. Additionally, we measured the impact of this correlation on outpatient visits for respiratory disease and quantified their interaction. Within this case-crossover study, we discovered cold waves and high- level PM_2.5_ were closely tied to increased outpatient visits for respiratory disease. Cold waves and PM_2.5_ exhibited a synergistic effect in precipitating these health issues. The independent effect of cold waves and the interaction between cold waves and PM_2.5_ on outpatient visits for respiratory disease decreased as the definition of cold waves became stricter and their duration extended. Comparative analyses over different periods have shown a decreasing trend in the independent effect of cold waves, while the synergistic effect with PM_2.5_ persisted. Vulnerability to cold waves and the synergistic effect were notably higher among males, children, and the elderly.

Currently, there are relatively few studies investigating the association between cold waves and outpatient visits for respiratory disease, with most research focusing on emergencies, hospitalizations, and deaths. The OR in this study was close to the estimated values in other studies, such as in Shanghai at 1.26 (95% CI: 1.14, 1.38) [37], Shanxi at 1.232 (1.090, 1.394) [23], Beijing at 1.811 (1.229–2.667) [38], and Nanjing at 1.54 (1.16, 2.04) [39]. These results indicate that the hazards of cold waves should receive sufficient attention. For instance, meteorological monitoring and early warning, as well as the maintenance of health services, are necessary. The health risks associated with cold waves and air pollution in high-altitude areas have not been sufficiently studied. A study on the impact of cold waves on the risk of population death in provincial capital cities of China revealed that the death risk in Xining city is much higher than in other western and same-latitude plain areas [22]. According to Tibet research, death risk rises with lower temperatures [40]. These studies emphasize that the health risks to the population from extreme weather events in high-altitude areas cannot be ignored. Some studies suggest that significant temperature differences between indoors and outdoors may exacerbate the risk of respiratory diseases [41, 42]. Notably, in Xining City, the heating period lasts for half a year, which may further amplify the risk of illness due to temperature differences. Comparisons of cold waves in different time periods show that the risk of respiratory disease generally decreases over time. This was attributed to the reduced frequency of cold waves after 2018, likely related to global warming [43, 44]. Comparing the interaction effects in different periods, the synergistic effect from 2019 to 2021 was found to be greater than that from 2016 to 2018, possibly due to the reduced individual effect of cold waves. Overall, extending the duration of cold wave responses through measures such as heating and keeping warm can effectively reduce the risk of illness and the onset of diseases [45, 46].

This study reveals that cold waves and PM_2.5_ significantly increase the risk of respiratory diseases, an effect closely related to physiological response changes caused by cold environments and air pollution. During cold waves, the stability of the atmospheric layer is enhanced, leading to an increase in the ground concentration of air pollutants such as PM_2.5_ [47], which can directly irritate the respiratory tract, trigger inflammatory responses, and potentially exacerbate existing respiratory diseases. Additionally, the cold environment may enhance the irritating effect of air pollutants on the respiratory tract, while the pollutants in the air may decrease the body’s adaptability to cold, significantly increasing the risk of respiratory diseases. Specifically, the inhalation of cold air directly cools the mucous membrane of the upper respiratory tract, causing vasoconstriction and mucous membrane dryness, leading to infections and inflammation [9, 48]. Furthermore, low temperatures and PM_2.5_ cause oxidative damage to bronchial epithelial cells, leading to bronchospasm and increased airway reactivity [49, 50], increasing the risk of diseases such as asthma. The cold environment also affects the immune system, reducing the activity of macrophages and lymphocytes, weakening the body’s ability to eliminate pathogens [51, 52]. PM_2.5_ can penetrate into the alveoli, directly suppressing the local immune response of lung immune cells [53, 54], making individuals more susceptible to pathogen invasion. Meanwhile, cold conditions and PM_2.5_ activate inflammatory cells in the respiratory tract, promoting the occurrence of inflammatory reactions [55, 56]. During cold waves, people often use heating devices, which may increase the concentration of harmful substances in indoor air (such as CO, PM_2.5_), exacerbating the exposure risk to the respiratory tract [57]. Cold air and poor air quality may limit outdoor activities, leading to indoor gatherings of people, thereby increasing the risk of respiratory diseases through airborne transmission. These findings highlight the importance of protecting respiratory health under conditions of cold and high-level of PM_2.5_, especially considering the cumulative negative impact on respiratory health of the interaction between cold waves and PM_2.5_. These results emphasize that the body may trigger a more intense physiological response under the combined effect of cold waves and air pollutants than under the influence of a single factor. This reveals the need to consider multiple impacts comprehensively and adopt more comprehensive and personalized health protection measures when facing these two interacting factors.

Stratified analysis shows that the risk of respiratory disease was slightly higher in males than females, but the difference was not significant. This could be related to factors such as males engaging more in outdoor work, greater temperature differences between indoor and outdoor environments, and demographic scale. Children and the elderly faced greater risks, which could be associated with physiological characteristics and the immune system [58–60]. This could also be linked to their simultaneous exposure to higher concentrations of PM_2.5_. The interaction between cold waves and PM_2.5_ shows statistically significant differences in the age groups of 15–64 and ≥ 65 years. Additionally, the physiological decline that often occurs with aging in the elderly may lead to a higher incidence of diseases. Therefore, exposure to cold waves and high levels of PM_2.5_ increases the risk of respiratory diseases in this group [60–62].

Although there is an increasing amount of research on the interactive effects of extreme temperatures and PM_2.5_ on mortality rates, the potential synergistic effects on outpatient visits for respiratory disease, especially during cold waves, have not yet been assessed. Our research presents new findings suggesting that the combination of cold waves and PM_2.5_ could have a synergistic effect, leading to respiratory health issues. The influence of these interactions decreased as the duration and intensity of cold waves increased, emphasizing the significance and possible advantages of reducing simultaneous exposure to cold waves and PM_2.5_, especially in high-altitude regions. Additionally, we found that combined exposure to cold waves and high level of PM_2.5_ can result in a 21.86% excess in respiratory disease outpatient visits. Hence, the implementation of efficient cold wave alerts, precautionary measures, and minimizing exposure to PM_2.5_ can yield significant benefits for public health.

This study has certain limitations. Firstly, the meteorological and pollution data primarily come from monitoring stations, not individual exposure data, which may lead to exposure errors. Secondly, our meteorological and PM_2.5_ data are based on outdoor measurements. In cold weather, most people spend more time indoors, and the impact of the indoor-outdoor temperature difference is overlooked. Thirdly, the study area is a high-altitude region with a unique climate, lower average temperatures, and a smaller population size, which limits the generalizability of our results to other areas.

## Conclusions

In this study, acute exposure to cold waves and PM_2.5_ was significantly associated with an increase in outpatient visits for respiratory disease, especially among children and the elderly. However, the risk impact of combined exposure to these two extreme conditions is greater than the sum of their individual effects. This emphasizes the public health importance of reducing particulate pollution when providing weather warning services to the public and highlights the potential health risks in high-altitude areas. Given the ongoing progression of climate change, regions like Qinghai Province on the plateau are sensitive to climate change and air pollution. With an expected increase in the frequency of extreme weather events and high pollution days, it is urgent to assess the health impacts of extreme weather events and air pollution.

### Electronic supplementary material

Below is the link to the electronic supplementary material.

Supplementary Material 1

## Data Availability

Data on outpatient visits cannot be published because they contain a great deal of personal information about the patients and their families. Please contact the corresponding author directly if you need anything else.

## Abbreviations

PM_2.5_: Fine particulate Matter
PM_10_: Inhalable particles
SO_2_: Sulfur Dioxide
CO: Carbon Monoxide
NO_2_: Nitrogen Dioxide
O_3_: Ozone
ICD-10: International Classification of Disorders, Tenth Revision
DLNM: Distributed lag non-linear model
Df: Degrees of freedom
REOI: Relative excess odds due to interaction
AP: Proportion attributable to interaction
S: Synergy index
