# Supplementary material for: Cold waves and fine particulate matter in high-altitude Chinese cities: assessing their interactive impact on outpatient visits for respiratory disease
Source: BMC Public Health. 2024 May 22;24:1377. doi: 10.1186/s12889-024-18896-x (PMC11110372; doi:10.1186/s12889-024-18896-x)

Supplementary Material

**Table S1** Daily meteorology, pollutants and outpatient visits for respiratory disease data for Xining from 2016 to 2021.

|  | Counts | Mean ± SD | min | median | max |
| --- | --- | --- | --- | --- | --- |
| Outpatient visits for respiratory disease | | | | | |
| Total | 393,185 | 179.4 ± 67.8 | 45 | 170 | 569 |
| Sex | | | | | |
| Male | 205,738 | 93.9 ± 34.9 | 22 | 86 | 294 |
| Female | 187,447 | 85.5 ± 34.5 | 15 | 81 | 275 |
| Age |  |  |  |  |  |
| 0-14 years | 167,914 | 76.6 ± 40.5 | 10 | 69 | 327 |
| 15-64 years | 187,432 | 85.5 ± 32.1 | 17 | 82 | 241 |
| ≥65 years | 37,839 | 17.3 ± 10.1 | 0 | 17 | 58 |
| Meteorological variables |  |  |  |  |  |
| Daily mean temperature (°C) | / | 6.4 ± 9.2 | -16.2 | 7.2 | 25.6 |
| Daily mean relative humidity (%) | / | 71.7 ± 16.2 | 15 | 57 | 94 |
| Air pollutants |  |  |  |  |  |
| PM2.5 (μg/m3) | / | 40.2 ± 27.9 | 4 | 33 | 392 |
| SO2 (μg/m3) | / | 20.0 ± 13.1 | 1 | 16 | 133 |
| NO2 (μg/m3) | / | 39.3 ± 16.1 | 1 | 36 | 110 |
| CO(mg/m3) | / | 1.4 ± 0.8 | 0.2 | 1.13 | 6.1 |
| O_3_(μg/m3) | / | 93.3 ± 33.4 | 11 | 92 | 281 |

**Table S2** Excess respiratory disease outpatient visits fraction and excess number of visits due to exposure to cold waves and PM_2.5_.

| Definition | Excess fraction | Excess number |
| --- | --- | --- |
| **Cold spell and low-level PM_2.5_** | | |
| 10th2D | 0.031 | 12,445 |
| 10th3D | 0.051 | 20,329 |
| 10th4D | 0.019 | 7,579 |
| 7.5th2D | 0.021 | 8,572 |
| 7.5th3D | 0.044 | 17,578 |
| 7.5th4D | 0.012 | 5,033 |
| 5th2D | 0.017 | 7,012 |
| 5th3D | 0.011 | 4,633 |
| 5th4D | 0.005 | 2,192 |
| 2.5th2D | 0.049 | 19,288 |
| 2.5th3D | 0.053 | 20,849 |
| 2.5th4D | 0.027 | 10,650 |
| **Non-cold spell and high-level PM_2.5_** | | |
| 10th2D | 0.015 | 6,288 |
| 10th3D | 0.018 | 7,412 |
| 10th4D | 0.019 | 7,498 |
| 7.5th2D | 0.02 | 8,096 |
| 7.5th3D | 0.025 | 9,898 |
| 7.5th4D | 0.025 | 10,045 |
| 5th2D | 0.027 | 10,684 |
| 5th3D | 0.026 | 10,278 |
| 5th4D | 0.029 | 11,627 |
| 2.5th2D | 0.025 | 10,195 |
| 2.5th3D | 0.027 | 10,904 |
| 2.5th4D | 0.029 | 11,602 |
| **Cold spell and high-level PM_2.5_** | | |
| 10th2D | 0.218 | 85,941 |
| 10th3D | 0.214 | 84,440 |
| 10th4D | 0.19 | 74,828 |
| 7.5th2D | 0.162 | 63,791 |
| 7.5th3D | 0.192 | 75,501 |
| 7.5th4D | 0.173 | 68,110 |
| 5th2D | 0.09 | 35,599 |
| 5th3D | 0.081 | 31,952 |
| 5th4D | 0.106 | 42,064 |
| 2.5th2D | 0.159 | 62,702 |
| 2.5th3D | 0.131 | 51,690 |
| 2.5th4D | 0.132 | 52,053 |

**Table S3** The number of days that the cold wave occurred under the definition of 7th2D to 7th4D.

| Years definitions | 7th2D | 7th3D | 7th4D |
| --- | --- | --- | --- |
| 2016 | 37 | 33 | 20 |
| 2017 | 21 | 14 | 14 |
| 2018 | 44 | 44 | 44 |
| 2019 | 22 | 20 | 10 |
| 2020 | 17 | 13 | 13 |
| 2021 | 22 | 18 | 18 |

**Figure S1** Spearman correlation between air pollutants and meteorological factors in 2016-2021 of Xining City, Qinghai Province, China. (*P < 0.05)


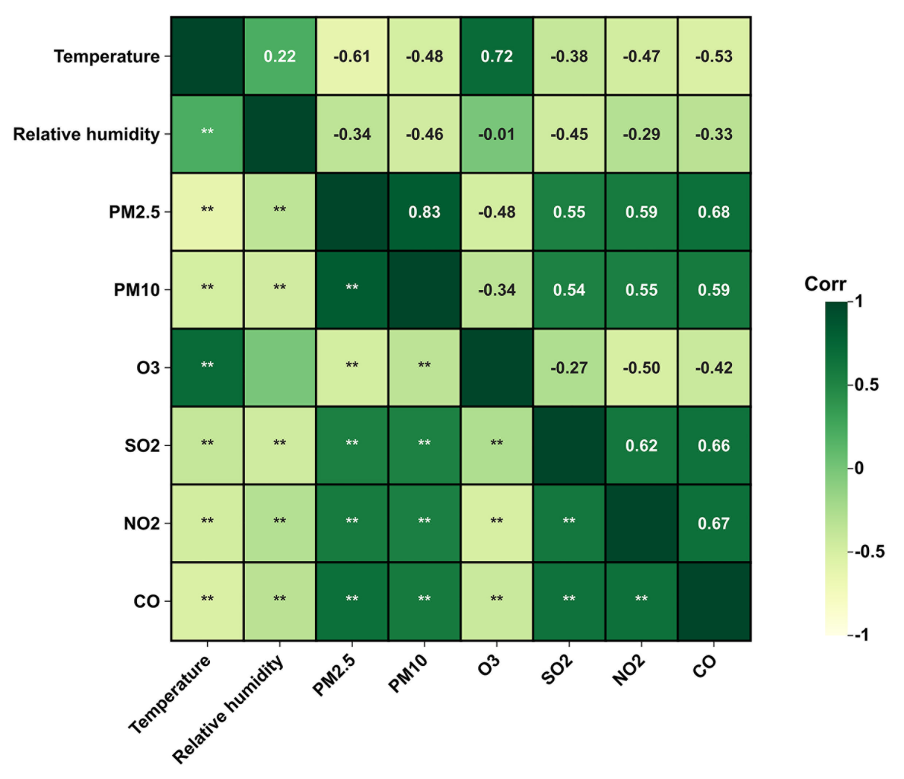


**Figure S2** OR of exposure to cold waves and PM_2.5_ associated with respiratory disease outpatient visits. (OR_10_ for cold wave and low-level of PM_2.5_; OR_01_ for non-cold wave and high-level of PM_2.5_; and OR_11_ for cold wave and high-level of PM_2.5_)





**Figure S3** Sensitivity analysis results. (Basic model refers to REOI from Octobe.r 15 through April 15, Multi-pollutant refers to combined air pollutants)





**Figure S4** Sensitivity analyses of the cross-base function in the model was “ns”.

**
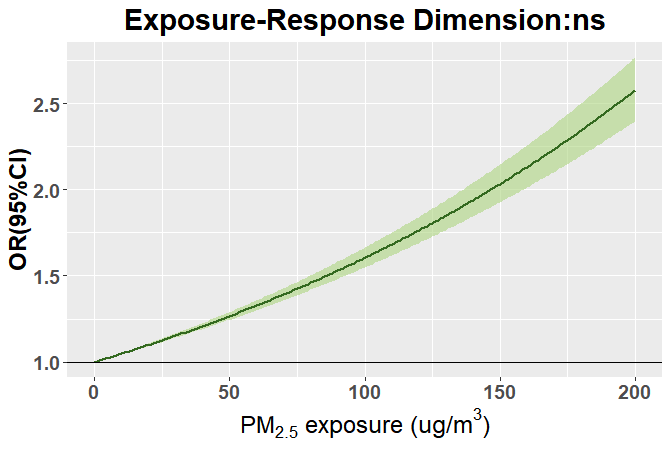
**

**Figure S5** Sensitivity analyses of the ORs of cold waves and PM2.5 for different time periods (2016-2018, 2016-2019, 2019-2021, 2020-2021).


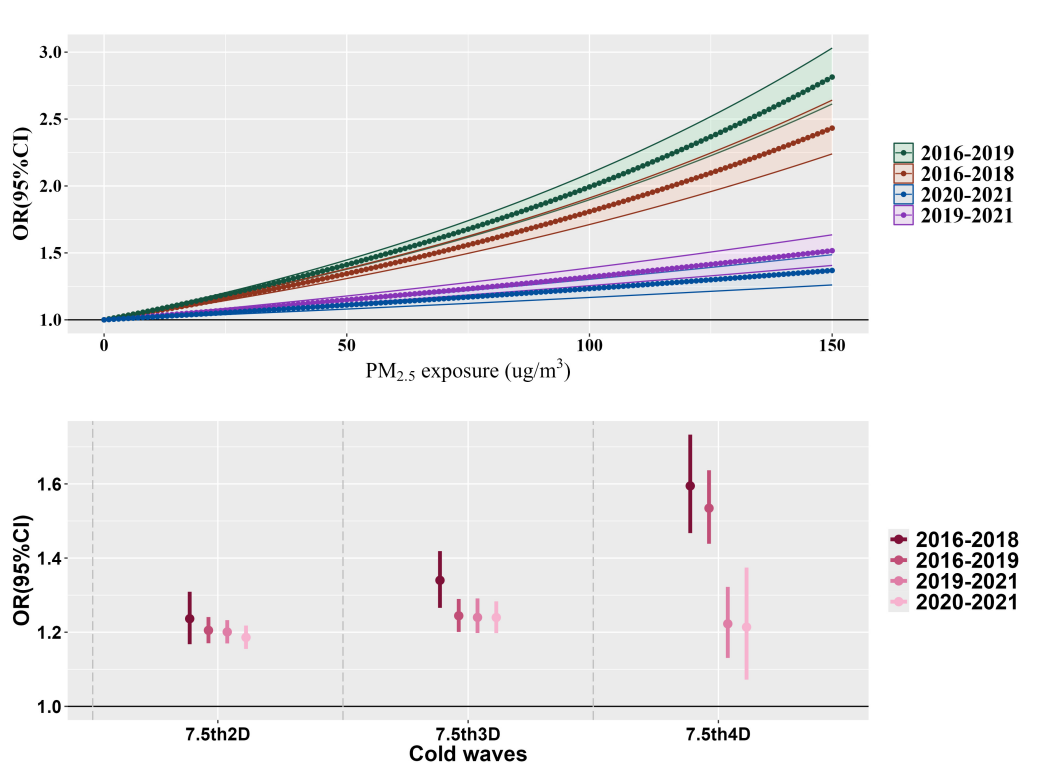

Supplement: Supplementary file 1 — Supplementary Material 1 [file 12889_2024_18896_MOESM1_ESM.docx]
